# Cognitive Profile in Ultra High Risk for Psychosis and Schizophrenia: A Comparison Using Coordinated Norms

**DOI:** 10.3389/fpsyt.2019.00695

**Published:** 2019-10-01

**Authors:** Liss Anda, Kolbjørn K. Brønnick, Jan Olav Johannessen, Inge Joa, Rune A. Kroken, Erik Johnsen, Maria Rettenbacher, Farivar Fathian, Else-Marie Løberg

**Affiliations:** ^1^TIPS Network for Clinical Psychosis Research, Stavanger University Hospital, Stavanger, Norway; ^2^Department of Biological and Medical Psychology, University of Bergen, Bergen, Norway; ^3^Division of Psychiatry, Haukeland University Hospital, Bergen, Norway; ^4^SESAM Centre for Age-Related Medicine, Stavanger University Hospital, Stavanger, Norway; ^5^Department of Public Health, Faculty of Health Sciences, University of Stavanger, Stavanger, Norway; ^6^Norwegian Centre for Mental Disorders Research, Haukeland University Hospital, Bergen, Norway; ^7^Department of Clinical Medicine, University of Bergen, Bergen, Norway; ^8^Medical University Innsbruck, Innsbruck, Austria; ^9^Outpatient Department, NKS Olaviken Gerontopsychiatric Hospital, Bergen, Norway; ^10^Department of Addiction Medicine, Haukeland University Hospital, Bergen, Norway; ^11^Department of Clinical Psychology, University of Bergen, Bergen, Norway

**Keywords:** at-mental-risk, psychosis, schizophrenia, prodromal, neurocognition, cognitive changes

## Abstract

**Background:** Cognitive impairment is not only a core aspect of schizophrenia but also commonly observed in help-seeking youth at ultra high risk for psychosis (UHR), with potential implications for prognosis and individualized treatment. However, there is no consensus on the cognitive profile in the UHR state, partly due to lack of valid comparisons of performance in established schizophrenia and UHR.

**Objectives:** To compare the cognitive functioning and profile of UHR subjects to a sample with schizophrenia, they were split into two groups based on duration of illness. Comparisons were made using coordinated norms based on healthy controls reflecting the younger UHR age spectrum.

**Methods:** Participants for UHR (*n* = 51) and schizophrenia groups (*n* = 19 and *n* = 22) were included from the Prevention of Psychosis and Bergen Psychosis 2 projects. All subjects completed a comprehensive neurocognitive test battery aiming to measure speed of processing, working memory, verbal learning, reasoning, and problem solving, as well as visual problem solving. Cognitive functioning was compared between groups based on coordinated norms using *z*-scores derived by regression modeling from an age-matched healthy control group (*n* = 61).

**Results:** UHR subjects showed significantly impaired speed of processing (*p* < 0.001) working memory (*p* = 0.042) and verbal learning, reasoning, and problem solving (*p* = 0.007) as compared to the control group. Visual problem-solving skills appeared unimpaired. UHR subjects significantly outperformed the schizophrenia group with duration of illness >3 years for speed of processing and working memory (both *p* < 0.001). There were no significant differences in performance between the UHR group and the group with duration of schizophrenia <3 years.

**Conclusion:** Cognitive performance is impaired in UHR subjects as compared to healthy controls and should thus be monitored when a person is deemed at high risk of psychotic illness. Spatial skills, as measured by tests using physical objects, appear less affected than other domains. The pattern of impairment is similar to that of a group with recent onset schizophrenia but is less severe than in a group with duration of illness <3 years.

## Introduction

Cognitive impairment is a core characteristic of schizophrenia (1). The majority of patients with schizophrenia fall below the cognitive performance level expected according to premorbid functioning or parental educational levels (2). Cognitive impairment likely precedes the appearance of overt psychotic symptoms, and some authors suggest that it is a neurobiological marker for psychosis risk (3). Impairment in episodic and working memory, speed of processing, verbal fluency, attention, and executive functions have robustly been demonstrated (4). Impairment in cognitive functioning is associated with a higher likelihood of relapse (5), poor functional outcome (6), and worse quality of life (7) and is thus an important prognostic indicator in clinical settings.

If cognitive decline starts in the prodromal phase of schizophrenia (3), it could be hypothesized that cognitive impairment also presents a challenge for help-seeking young people at ultra high risk for psychosis (UHR). Cognitive impairment in UHR could affect both social and academic functioning as well as the ability to profit from psychosocial therapy and interventions, which are often the first choice for this patient group (8). Managing or ameliorating cognitive dysfunction in psychotic disorders is therefore central to clinical recovery and to helping people function in the community. In addition, understanding how UHR cognitive changes compare to those seen in full-blown psychosis may further the understanding of etiology and course in psychotic disorders.

### Cognitive Impairment in the Psychosis Continuum

The continuum model of psychosis sees the psychosis spectrum as ranging from mild attenuated experiences in many otherwise healthy individuals, to clinically significant and severe symptoms in a few who fulfill diagnostic criteria for psychotic disorders (9). The UHR state falls into the milder end of this continuum, while schizophrenia is considered to be the most severe form in terms of symptom load and duration (10). In accordance with this, cognitive impairment in UHR appears to be milder than in schizophrenia. Furthermore, impairment in first-episode psychosis has been found to be less severe than in chronic schizophrenia (11). Alongside studies showing slightly greater impairment in persons with a longer duration of untreated psychosis, this might suggest that abnormal neurodevelopmental processes happen prodromally and continue after the onset of psychosis (12).

A meta-analysis found 20% (confidence interval, 17–25%) of UHR patients to develop full-blown psychosis (13, 14). UHR individuals often seek help because psychosis-related symptoms and signs cause functional decline and reduced quality of life. Although up to 65% of UHR subjects clinically recover from attenuated psychosis symptoms within 2 years (15), they often continue to report other mental health problems, which require targeted intervention (16). Early intervention services aimed at reducing the duration of untreated psychotic symptoms may thus improve outcome in those at risk for psychosis (17) regardless of conversion status by paving the way for tailored support.

Understanding the cognitive profile of UHR as compared to general population controls and patients with schizophrenia may improve service delivery and individualized treatment and prognosis prediction for this group, potentially preventing psychosis conversion in some cases. Cognitive changes seem to predict conversion to psychosis in UHR individuals, with longitudinal studies indicating worsening cognitive functioning as an early sign of eventual full-blown psychosis (18). Distractibility has also been shown to predict the UHR marker of voice hearing in adolescents (19), indicating an association between cognitive functioning and psychosis symptoms.

Cognitive screening may contribute meaningfully to efforts to individualize early intervention services aimed at supporting UHR youth. Cognitive remediation training may improve cognitive functioning (20) and has few, if any, negative side effects, making it appropriate for UHR groups (21). Although the effect on symptom load appears to be marginal (22), improved cognition might improve function (23). Handling cognitive challenges is therefore important both for UHR patients who develop full-blown psychosis and to the large subgroup whose attenuated symptoms do not worsen, but for whom impaired cognitive functioning may still remain a problem (24). However, we cannot successfully provide this without more detailed knowledge about the nature of the cognitive challenges of this group.

### UHR Groups and Cognitive Changes in Psychosis

It has been suggested that cognitive performance in UHR groups lie somewhere in between that of healthy controls and established psychotic illness (25). However, there is no clear consensus on the trajectory of cognitive changes ahead of and during psychotic illness. Subjects who later transit to psychosis have been found to show greater deficits than those who do not, albeit with modest effect sizes (26–28). These differences are not merely due to general cognitive ability (27). While some have argued that cognition declines across the course of psychotic illness (29), others have found no evidence of cognitive decline in patients with UHR, noting that cognitive performances improve at follow-up (28). Our own research group similarly found significant cognitive improvement across the acute phase of psychosis (30). A recent 24-month longitudinal study of young people with early onset schizophrenia also found this group to have a similar cognitive course to healthy controls, albeit functioning at an overall lower level (31). Improvement has also been seen in first episode schizophrenia, even with test batteries designed to withstand learning effects (32). The only study to date to retest UHR subjects after 10 years found no decline in cognition except in tests of immediate verbal learning and memory (33). This study also found cognitive change over the decade not to be related to baseline IQ, symptomatic change, or transition status.

There is a similar lack of consensus on changes in the UHR phase in relation to individual cognitive domains. In their 2014 meta-analysis, Bora et al. found the greatest impairment across UHR groups in symbol coding tests and more general measures of visuospatial working memory (28). A meta-analysis by Fusar-Poli, Deste (26), however, found significantly lower general intelligence in subjects deemed at high risk of psychosis, with verbal and visual memory most impaired. They found no group differences in overall speed of processing, although also they noted that the digit-symbol coding task was the single test showing the biggest discrepancy between high-risk subjects and healthy controls. The notable variability in previous findings may be partly due to measurement discrepancies across studies. In addition, a variety of test batteries have been used. The UHR group is also clinically and demographically diverse. Getting a representative sample may be affected, e.g., by restricted access to early intervention services or mental health care in the area of recruitment. Comparing data from UHR groups to those from groups with psychosis also remains difficult due to the young age of the UHR population, which means that coordinated norms do not exist for many commonly used tests of cognitive functioning.

We designed the present study aiming to overcome challenges associated with young subject age using norms based on regression analyses of a control group sample, in order to allow for comparison with younger subjects across tests while adjusting for age and sex, thus enabling a comparison of cognitive profiles in UHR and schizophrenia. We also included both UHR subjects and two comparison schizophrenia groups from similar catchment areas, with universal access to free health care and well-structured clinical practices for early intervention in psychosis, as we aimed to get a more representative view of UHR cognitive performance than would be allowed when recruiting in a more restricted public health care system.

### Aims and Hypotheses

The aim of the current paper is to examine the nature of cognitive dysfunction in UHR at the time of help seeking. UHR sample performance will be compared to performance in two groups with schizophrenia: one with recent onset of illness and one with longer duration of schizophrenia. Comparisons will be based on norms derived from healthy controls reflecting the younger age spectrum. These comparison groups will also allow us to use cognitive performance to illuminate the continuum model of psychotic illness.

We hypothesize the UHR group performance to fall below that of the healthy controls but above that of the schizophrenia groups. A secondary hypothesis is that the performance of recent onset schizophrenia participants will fall between the performance of the UHR group and the group with longer duration of illness. Given that measures of working memory and processing speed are found to be significantly impaired across studies, we hypothesize that impairment especially will be evident in demanding tasks loading on these cognitive functions, with our tests specifically designed to explore this.

## Methods

### Study Design

UHR subjects were included from the Prevention of Psychosis Project (POP), an early intervention and treatment study encouraging at-risk individuals through information campaigns to seek relevant and early professional support. Rolling inclusion of POP participants took place from March 2012 to December 2019 in health-care regions Stavanger and Fonna in Norway, with subjects assessed by low-threshold detection teams. POP offered participants a multimodal treatment program, adding antipsychotic medication at imminent risk of conversion only. The project aimed to significantly reduce the proportion of high-risk subjects whom convert to psychosis in the catchment areas and has been described in detail elsewhere (34). Participants in the current substudy were recruited between 2012 and 2016.

Schizophrenia subjects were drawn from the Bergen Psychosis Project 2 (BP2). BP2 consists of a pharmaceutical-industry-independent international and multisite pragmatic, randomized-controlled trial (RCT) comparing three antipsychotics (amisulpride, aripiprazole, and olanzapine) for effects and side effects, with the aim of improving the specificity of antipsychotic treatment. An observational cohort of patients with psychosis not eligible for the RCT was included for comparison. All participants were followed up for 12 months. Participants in the current substudy were recruited between 2013 and 2016 from hospital sites in Bergen, Stavanger, and Trondheim in Norway, as well as from Innsbruck, Austria.

### Participants

#### UHR Subjects (N = 52)

Inclusion criteria for participants drawn from the POP project were age 13–65 years and meeting diagnostic criteria for prodromal syndrome according to the Structured Interview For Prodromal Syndromes (SIPS) (35). The SIPS describes three different ways of fulfilling prodromal syndrome criteria. These are as follows: (1) brief intermittent psychotic syndrome (BIPS) as defined by experience of frank psychotic symptoms scoring at least 6 on the SOPS scale at least once per month but only in the last 3 months. brief intermittent psychotic syndrome is separated from current psychotic disorder by frequency and duration/urgency. (2) Attenuated positive symptoms syndrome, meaning recent experience of attenuated positive symptoms scored to 3–5 on the scales P1–P5 of SOPS, starting or worsening in the past 12 months and occurring at least once per month. (3) Genetic risk and deterioration syndrome, defined by a combined first-degree family history of nonaffective psychotic disorder and a 30% or greater estimated drop in function as measured by Global Assessment of Functioning score over the past 12 months. In addition to meeting prodromal syndromes criteria, participants were also required to have IQ ≥ 70, ability to understand and speak Norwegian, and ability to understand and sign an informed consent or assent for minors’ document. Exclusion criteria were any current or lifetime psychotic disorder, if symptoms were better accounted for by an axis I, axis II, or substance use disorder, with the exception of schizotypal personality disorder, lifetime use of antipsychotic medication exceeding 4 weeks, or any known neurological or endocrine disorders that may have caused the presented psychotic symptoms. They were also required to not be using nor having used any antipsychotic medication (regardless of dosage) for more than 4 weeks lifetime.

#### Subjects With Schizophrenia (N = 48)

Subjects were included from the BP2 project if they had completed the comprehensive neuropsychological assessment forming part of their 3-month follow-up. Inclusion criteria for the RCT part of the BP2 were age >18, active psychosis as determined by a score ≥4 on either of the Positive and Negative Symptoms Scale (PANSS) interview (36) items for delusions (P1), hallucinatory behavior (P3), grandiosity (P5), suspiciousness/persecution (P6), or unusual thought content (G9); no known neurological or endocrine disorders likely to have caused the presented psychotic symptoms and the ability to understand and speak the site native language (in Norway or Austria). Inclusion criteria for the observational cohort part of the BP2 were age >16 and previous or current psychosis. Diagnoses were determined by the Structured Clinical Interview for the DSM-IV Axis I Disorders (SCID I) (37), with the following ICD-10 disorders eligible for participation in the BP2 study: schizophrenia (F20), schizotypal disorder (F21), delusional disorder (F22), acute psychotic disorders (F23), schizoaffective disorder (F25), other organic psychotic disorders (F28), and unspecified nonorganic psychosis (F29). The current sub-study only included BP2 participants fulfilling criteria for F20 schizophrenia to ensure a more homogenous comparison group in relation to cognitive performance.

#### Healthy Control Subjects (N = 61)

Healthy controls were recruited at the Stavanger site as part of both POP and BP2 projects. They were recruited among Stavanger University Hospital employees and their networks, high schools in the local area and posters in social security offices (NAV), aiming to gender and age match UHR participants and cover the age range of both UHR and psychosis participant groups. Participants with a known first-degree family history of psychiatric disorder or current or past drug dependence (other than nicotine products) were excluded from participating. All healthy controls received a small payment to cover travel costs and time.

### Symptom and Functional Level Measures

UHR group baseline symptom load was measured by the SIPS interview (35). BP2 participants completed a PANSS assessment at baseline. A Structured Clinical Interview for the DSM-IV Axis I Disorders interview was administered to both groups by trained clinicians in order to determine diagnoses, with detailed results displayed in **Table 1**. Functioning was measured for all participants by the Global Assessment of Functioning, split version (38). All UHR subjects were antipsychotic naive (as per inclusion criteria) at the time of testing. Antipsychotic drug use in the schizophrenia group with psychosis was converted to defined daily doses (DDD), with DDD defined as “the assumed average maintenance dose per day for a drug used for its main indication in adults” (39). The control group completed PQ21 as well as a MINI assessment in order to exclude any subjects with subthreshold psychotic symptoms or other mental illness. However, no control group participant was excluded due to this.

**Table 1:** Demographic variables and baseline cognitive test scores by group.

	UHR (*n* = 51)	Schizophrenia duration <3 years (*n* = 19)	Schizophrenia duration <3 years (*n* = 22)	Controls (n = 61)
Mean age (SD)	17.0 (2.9)	27.0	33.2	23.9 (10.8)
Female %	61.2	21.1	59.1	55.7
**Measures of function**				
Baseline GAF^1^ functioning (SD)	49.4 (13.3)	47.4	46.3	88.6 (4.82)
Baseline GAF1 symptoms (SD)	45.7 (8.4)	47.6	47.7	87.0 (5.96)
PAS2 Childhood social functioning mean (SD)	1.47 (1.27)	N/A	N/A	N/A
PAS^2^ Childhood scholastic performance mean (SD)	2.41 (1.34)	N/A	N/A	N/A
PAS^2^ Childhood adaptation to school mean (SD)	1.16 (1.10)	N/A	N/A	N/A
PAS^2^ Early adolescence social functioning mean (SD)	1.70 (1.38)	N/A	N/A	N/A
PAS^2^ Early adolescence scholastic performance mean (SD)	2.55 (1.37)	N/A	N/A	N/A
PAS^2^ Early adolescence adaptation to school mean (SD)	1.80 (1.36)	N/A	N/A	N/A
**Symptoms and clinical assessments**				
SCID^3^ diagnoses				
Schizophrenia F20.x		19	22	
Bipolar disorder II F31.x	1			
Depressive disorder F32.x-33.x	12			
Dysthymia F34.x	1			
Anxiety disorders F41.x	7			
OCD F42.x	1			
PTSD F43.1	2			
Adjustment disorder F43.2	1			
Somatoform disorder F45.x	1			
Substance use disorders F10.x-19.x	2			
Psychiatric disorder NOS F99.x	1			
Psychotic disorder NOS F29.x	3			
No diagnosis	17			
Mean age of onset for psychosis (SD)	N/A	25.7 (5.9)	22.6 (11.5)	
Mean years duration of illness (SD)	N/A	1.3 (0.85)	10.6 (8.5)	
AD medication naïve at baseline %	100	36.8	22.7	
DDD mean (SD)	N/A	1.8(0.6)	1.4 (0.7)	
PANSS4 positive mean (SD)	N/A	19.8 (3.7)	21.3 (4.0)	
PANSS^4^ negative mean (SD)	N/A	21.1 (6.2)	18.0 (6.5)	
PANSS^4^ general mean (SD)	N/A	39.1 (7.8)	38.9 (8.4)	
SIPS5 positive mean (SD)	10.7 (3.3)	N/A	N/A	
SIPS^5^ negative mean (SD)	11.5 (6.1)	N/A	N/A	
SIPS^5^ general mean (SD)	8.9 (3.6)	N/A	N/A	
**Cognitive composites z-score group means (SD)**				
Speed of processing^6^	−0.78 (0.89)	−0.97 (1.41)	−1.80 (1.13)	
Working memory^7^	−0.51 (0.82)	−0.18 (1.08)	−1.72 (1.66)	
Verbal learning, reasoning and problem solving^8^	−0.50 (0.65)	−0.59 (0.78)	−0.37 (1.12)	
Visual problem solving^9^	−0.12 (1.18)	−0.43 (1.17)	−0.40 (1.19)	

^1^GAF; Global Assessment of Functioning, 2PAS, Premorbid Adjustment Scale; ^3^SCID, Structured Clinical Interview for DSM-IV; ^4^PANSS, Positive and negative symptoms scale; 5SIPS, Structured Interview for Prodromal Symptoms; ^6^Trailmaking A, WAIS coding, CWIT color, and word reading; ^7^Trailmaking B, WAIS number span and letter number sequencing, inhibition, and inhibition and switching conditions from D-KEFS CWIT; ^8^D-KEFS FAS, WAIS vocabulary, and CVLT; ^9^WAIS block design and WMS spatial span.

UHR subjects also completed a Norwegian version of the Premorbid Adjustment Scale (PAS) (40), a structured interview aiming to retrospectively assess social and academic premorbid adjustment. PAS yields five subscales used in the present study: Sociability and Withdrawal, Peer Relationships, Academic Achievement, Adaptation to School and Ability to Form Interpersonal and Sexual Relationships, each assessed for childhood (11 and younger), early and late adolescence (12–15 and 16–18 years), and adulthood (19 and older). For the purposes of simplified reporting, a mean social adjustment score was calculated by averaging the scores of Sociability and Withdrawal and Peer Relationships for each stage.

### Cognitive Measures

A comprehensive neurocognitive test battery was administered to POP project UHR participants at baseline and to BP2 participants and healthy controls at their 3-month follow-up. The battery was designed to assess verbal functioning, visuo-spatial functioning, and executive functions. Tests included in the present study were Trail Making A (TMA) and Trail Making B (TMB) (41), the California Verbal Learning test (42), Wechsler Adult Intelligence Scale III tests for number span, letter number sequencing, vocabulary, and block design (WMS) (43), WMS spatial span (44), as well as Delis–Kaplan Executive Function System (D-KEFS) Color Word Interference Test (CWIT), and FAS verbal fluency tests (45). Trained staff administered all neurocognitive testing.

### Statistical Analysis

All analyses were performed using the Statistical Package for the Social Sciences 25.0.

#### Calculation of Coordinated Norms

Neuropsychological score variables were assessed for normality by way of creating histograms for inspection, both overall and within groups. We standardized test raw scores by calculating *z* values relative to the baseline performance of our group of healthy controls. Calculation of *z*-scores was done to allow for comparisons across domains and tests and also because the young age of the UHR group in particular meant that valid test norms do not exist. This was done by running linear regression analyses for each variable, using age and gender as predictor variables. Based on the results from these we calculated expected scores adjusted for age and gender for each subject. Individual subject *z*-scores were then calculated by subtracting the expected score from the observed score in each variable and then dividing by the standard deviation of the control group. For tests where a higher raw score indicates worse performance (TMA, TMB, and CWIT), *z*-scores were inverted before further analyses. All negative *z*-scores thus indicate performance below that of controls.

#### Calculation of Cognitive Profile

For the purposes of the current study, test results were selected and grouped into cognitive domains according to existing literature and neuropsychological conventions (46). The cognitive domains used for the purposes of this study were speed of processing (TMA, WAIS digit symbol coding, CWIT color and word reading conditions), working memory (TMB, WAIS number span and letter number sequencing, inhibition, and inhibition and switching conditions from D-KEFS CWIT), verbal learning, reasoning, and problem solving (D-KEFS FAS, WAIS vocabulary, and CVLT), and spatial reasoning (WAIS block design and WMS spatial span). The calculated mean *z*-score for the tests comprising each domain formed the score for each of these four domains.

#### ANOVA Group Comparison

Cognitive subscale *z*-scores were compared between the groups using one-way ANOVAs. The four comparison groups were UHR participants and two F20 groups, the first comprising participants with duration of illness up to 3 years (SZ1) and the second group with duration extending 3 years (SZ2), as well as healthy controls. Levene’s test was used to check equality of variances between groups. Where the *F* value indicated significant between-group differences, post-hoc pairwise *t* tests were performed in order to assess these. The Siddaq correction was applied to adjust for multiple comparisons.

#### Correlational Analysis

We calculated Pearson correlations between the cognitive subscales and symptom scores, i.e., SIPS and PANSS scores, respectively, for the UHR and F20 groups. We also calculated the correlation between cognitive performance and antipsychotics DDD as well as duration of illness for the F20 groups.

## Results

Demographic variables for all three groups are displayed in **Table 1**. Owing to the young mean age of the UHR and healthy control groups (as young as 13, with many still living at home and in full-time mandatory education), years of education, living status, and employment levels were not compared between groups.

### Neuropsychological Profile and Between-Group Differences

**Figure 1:**
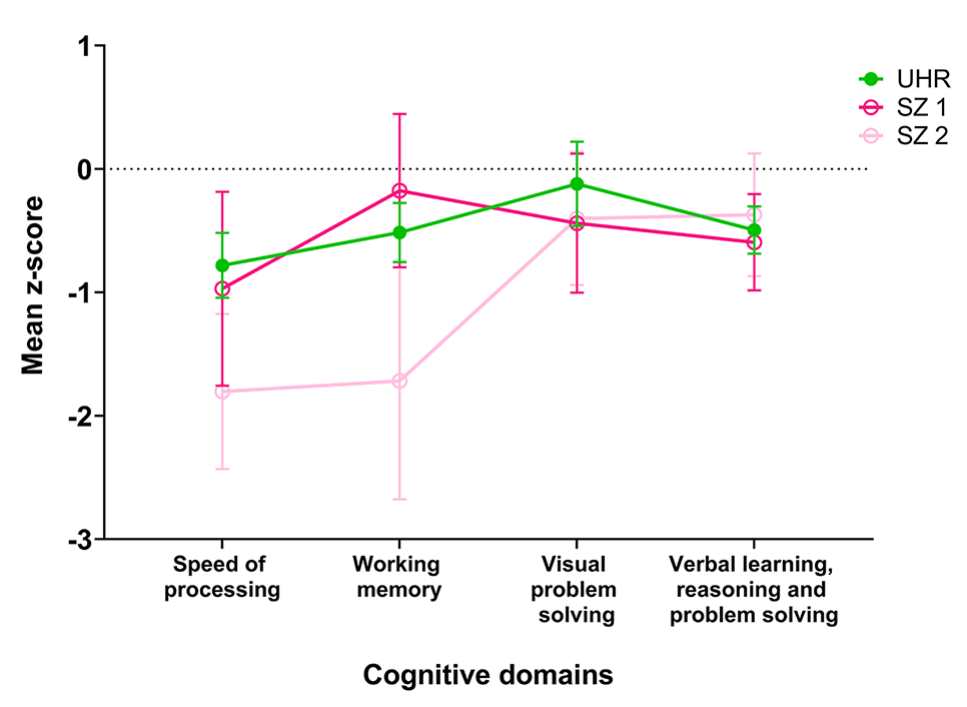
Cognitive profiles in UHR and groups with recent onset and longer duration schizophrenia. UHR, ultra high risk group; SZ1, group with recent onset schizophrenia (< 3); SZ2, group with remote onset schizophrenia (> 3 years). Error bars show 95% CI.

A one-way ANOVA of *z*-scores based on age- and gender-controlled norms revealed significant differences in cognitive performance between groups, with both the schizophrenia and UHR groups scoring lower than healthy controls. Between-group differences were significant for speed of processing [*F*(3) = 18.24, *p* < 0.001], working memory [*F*(3) = 13.71, *p* < 0.001), and verbal learning, reasoning, and problem solving [*F*(3) = 4.94, *p* = 0.003), but not for visual problem solving [*F*(3) = 1.16, *p* = 0.327]. Group cognitive profiles are displayed for comparison in **Figure 1**. The UHR group had significantly lower scores than the control group for speed of processing (*p* < 0.001), working memory (*p* = 0.042), and verbal learning, reasoning, and problem solving (*p* = 0.007). They scored significantly better than the longer duration of illness schizophrenia group for speed of processing and working memory (both *p* < 0.001). There were no significant differences in performance between the UHR group and the recent onset schizophrenia group in any domain. For complete results of pairwise comparisons, please refer to **Table 2**.

**Table 2:** Cognitive domains *z*-score comparisons between UHR, schizophrenia, and control groups.

Cognitive domain	*F* (sig)	*p* (UHR vs. SCZ1)	*p* (UHR vs. SCZ2)	*p* (UHR vs. CTR)	*p* (SCZ1 vs. SCZ2)	*p* (SCZ1 vs. CTR)	*p* (SCZ2 vs. CTR)
Speed of processing^1^	18.24 (<0.001)	0.981	<0.001**	<0.001**	0.080	0.002*	<0.001**
Working memory^2^	13.71 (<0.001)	0.770	<0.001**	0.042*	<0.001**	0.995	<0.001**
Verbal learning, reasoning, and problem solving^3^	4.944 (0.003)	0.998	0.998	0.007**	0.022	0.027*	0.303
Visual problem solving^4^	1.27 (0.327)	0.840	0.890	0.998	1.000	0.587	0.652

*p < 0.05, two-tailed. **p < 0.001, two-tailed UHR, ultra high-risk group; SCZ1, recent onset schizophrenia group; SCZ2, longer duration schizophrenia group; CTR, control group.

^1^ Trailmaking A, WAIS coding, CWIT color, and word reading; ^2^ Trailmaking B, WAIS number span and letter number sequencing, inhibition, and inhibition and switching conditions from D-KEFS CWIT; ^3^ D-KEFS FAS, WAIS vocabulary and CVLT; ^4^ WAIS block design and WMS spatial span.

### Correlational Analysis

Working memory performance was correlated with SIPS positive symptoms for the UHR group, while PANSS negative score was significantly correlated with verbal learning for participants with schizophrenia. However, neither of these correlations remained significant upon applying a Bonferroni correction for multiple comparisons. There were no significant correlations between DDD and cognitive domain scores. Complete results from correlation analyses are displayed in **Table 3**.

**Table 3:** Correlations between cognitive domains and clinical variables.

	Speed of processing^3^ Pearson correlation (*p*)	Working memory^4^ Pearson correlation (*p*)	Visual problem solving^5^ Pearson correlation (*p*)	Verbal learning, reasoning and problem solving^6^ Pearson correlation (*p*)
**UHR participants**				
SIPS positive score^1^	−0.165 (0.278)	−0.305 (0.039)*	−0.134 (0.374)	−0.063 (0.682)
SIPS negative score^1^	0.141 (0.372)	0.137 (0.383)	0.083 (0.597)	−0.025 (0.875)
SIPS general score^1^	0.095 (0.544)	0.101 (0.514)	−0.048 (0.755)	0.175 (0.262)
**F20 participants**				
PANSS positive score^2^	0.101 (0.594)	−0.015 (0.938)	0.086 (0.597)	0.127 (0.434)
PANSS Negative score^2^	−0.043 (0.820)	0.117 (0.554)	−0.260 (0.105)	−0.374 (0.017)*
PANSS General score^2^	−0.086 (0.652)	−0.096 (0.626)	−0.275 (0.086)	−0.192 (0.234)
Antipsycotic_DDD	−0.071 (0.743)	−0.138 (0.540)	−0.123 (0.508)	−0.179 (0.336)

*p < 0.05, two-tailed. *^1^* *SIPS, Structured Interview for Prodromal Symptoms;* *^2^* *PANSS, Positive and Negative Symptoms Scale;* *^3^* *Trailmaking A, WAIS coding, CWIT color, and word reading;* *^4^* *Trailmaking B, WAIS number span and letter number sequencing, inhibition, and inhibition and switching conditions from D-KEFS CWIT;* *^5^* *D-KEFS FAS, WAIS vocabulary and CVLT;* *^6^* *WAIS block design and WMS spatial span.*

## Discussion

UHR subjects performed significantly worse than the healthy control group on measures of speed of processing, cognitive flexibility, and verbal learning, reasoning, and problem solving. This is in line with our hypothesis and confirms cognitive impairment as an observable early sign of a potential psychotic disorder. The UHR group also outperformed the schizophrenia group with duration of illness longer than 3 years on speed of processing and working memory. There were notably no significant differences in performance between the UHR group and the recent onset F20 group. The cognitive performance profile of the UHR group fell in between that of the longer duration schizophrenia group and that of the healthy controls while matching that of the recent onset participants. Our results support previous meta-analytic and review findings (26, 47) of impaired cognitive performance in UHR groups, with authors arguing for this as a measurable potential vulnerability marker preceding severe positive symptoms (3). Interestingly, the UHR group performance in tests of spatial abilities (WAIS block design and WMS spatial span) was almost identical to that of the control group. Our findings are similar to those of another recent study which found no impairment in visual skills in a clinical high-risk group, as opposed to in all other domains (48). Spatial abilities have previously been found to discriminate between UHR individuals who convert to psychosis and those who do not (49, 50). Others have found no such link (51, 52), although spatial abilities were found to be impaired. One study also found a link between spatial span and functional outcome (52), meaning that these skills could be important when working to limit functional loss in UHR individuals.

We suggest that the unimpaired spatial performance of the UHR sample in our study could, in part, be due to the inclusion of WAIS block design and WMS spatial span tests, both of which use physical test objects. Many studies have mainly used nontactile, screen-based tests, such as the delayed response task, rather than tests involving physical objects. Screen-based tests are likely to load more heavily on the working memory and visuospatial sketchpad aspect of spatial skills than do tests supported by physical objects. If so, this could indicate that the inclusion of physical objects may aid the cognitive functioning of this group. We have been unable to find any previous research directly comparing these two aspects of visuospatial functioning in UHR groups.

SZ1 group performance equaled that of the UHR participants. This was surprising, as we were expecting the UHR group to outperform SZ1. Significant differences were neither found in DDD of medication received nor in baseline antipsychotic naivete between the two F20 groups. The similarity to UHR performance in the SZ1 group might reflect the findings of other papers, which have found few significant performance differences between UHR and first-episode psychosis groups while seeing greater impairments in more chronic schizophrenia (11). However, we hesitate to read too much into these results, as statistical power was low given the small subgroup size.

The significant difference in performance between the SZ1 and SZ2 groups might be explained in several different ways. Cognitive performance may decline over the course of illness with active psychosis being a neurotoxic state (4), although several authors have refuted this idea (12, 28). Another explanation may be that better cognitive performance equips people to better take advantage of and adhere to any treatment offered, thus aiding a quicker recovery. A final explanation which seems likely is that the recent onset SZ1 group is genetically diverse. It plausibly includes people likely to develop both chronic and less severe courses of illness, with better cognitive functioning as characteristic of those more likely to recover. Ultimately, only longitudinal studies, preferably also tracking genetic factors (53), may explain this pattern.

Our results lend support to previous findings that speed of processing, working memory, and verbal ability show particular impairment studies in both UHR and psychotic disorder groups (4). Tasks requiring speed and cognitive flexibility in manipulating information appear to present difficulties for both UHR and schizophrenia groups. Previous functional MRI studies have found changes in major associative fiber tracts/functional connectivity in UHR groups (54). DTI studies have found reductions in fractional anisotropy as well as increased diffusivity (55) in the UHR phase, indicating both demyelination and deterioration in the axonal membrane. Taken together, these studies suggest reduced connectivity in UHR. Our findings of impaired cognitive flexibility and speed can be consistent with reduced white matter connectivity, perhaps to a lesser extent than in schizophrenia given the higher speed of processing of UHR individuals. In line with this, reductions in white matter and abnormalities in white matter microstructure in prefrontal and temporal lobe areas have been found to be more pronounced in schizophrenia than in UHR groups.

Our findings highlight the importance of clinical attention to cognitive problems when aiming to alleviate distress in the UHR patient group. The cognitive impairment reported by our study suggest cognitive domains that should be targeted in the UHR groups in, e.g., academic settings. Working memory, attention, and speed of processing performance have been found to predict over half of the variance over time in school or work participation in clinically stable first-episode psychosis (56). A UHR treatment approach including focused interventions for cognitive deficits, such as psychoeducation, cognitive training, and physical exercise (57), may therefore be crucial in preventing further functional loss over time. The relationship between social and cognitive functioning would also be an interesting topic for future research.

Abnormal synaptic pruning in UHR groups (58) might explain their impaired speed of processing. Longitudinal research in healthy children has found a puberty-related dip in performance speed in tasks requiring working memory and decision making, linked by authors to normal synaptic proliferation at this stage (59). The same authors argue that healthy synaptic pruning after puberty ensures the more effective cognitive performance seen their young adult comparison sample. Any disturbance to this pruning process is likely to underpin cognitive impairment in UHR and schizophrenia when compared to healthy adults. Future research should investigate any associations between such pruning disturbances and not only cognitive impairment but also cognitive change and potential growth in UHR groups.

Despite the clinical diversity of the UHR group, the variance in performance is much greater in the schizophrenia group. One major reason for this is likely that they simply are a more diverse group. First of all, variance is to be expected in a group spanning all of adolescence in age. Second, the majority of our UHR sample will most likely not go on to develop full-blown psychosis. As with all cross-sectional UHR studies, our sample thus includes a number of false positives. Our results must therefore be interpreted with some caution when searching to elucidate the trajectory of cognitive changes in the UHR subgroup who go on to develop psychotic disorders. However, our findings are still able to inform clinical work with UHR groups, as our sample’s diversity is representative of the variability inevitably seen in these patients.

Another possible contributor to the greater variability in the UHR group is that several different trajectories exist within the development of cognitive impairment between the stages of UHR to full-blown psychosis. It is also possible and likely that different aspects of cognition develop differently. As noted by Corigliano et al. (11), cross-sectional group data also mask potential differences between individual trajectories of cognitive functioning, where people may both improve or worsen, as well as remaining stable. It is also possible that better cognitive performance at the UHR stage may ameliorate the course of illness by better absorbing any ensuing decrement in cognitive function as well as by improving the individual’s ability to make use of any help and treatment offered. Worse performance in chronically ill groups might thus imply lower baseline functioning rather than an ongoing decline. Last, poor cognitive functioning in UHR may, to some degree, independently coexist with psychosis-like experiences. This idea is supported by the existence of some cognitive deficits in UHR individuals who do not develop psychotic disorder (60). Further longitudinal work is required to identify the path of each UHR person, to reveal any individual or group patterns of change. Our findings indicate that a decrement in cognitive functioning is in place and measurable before the appearance of clinically significant positive symptoms of psychosis. Cognitive impairment also appears to be more severe in participants with established illness. Although our cross-sectional design precludes us from concluding firmly, this may indicate that further cognitive changes take place during the transition from prodromal symptoms to full-blown psychosis. Previous work from our research group found cognitive improvement during the early treatment of a psychotic episode (30). However, the present study reinforces the fact that, despite this improvement, the decrement in functioning remains in schizophrenia even after the acute phase. Although not every UHR participant will develop psychotic disorder, our current findings indicate that cognition most likely continues to change between the UHR stage and established psychotic disorder, in line with a neurodevelopmental but not necessarily uniformly degenerative model of psychosis.

## Limitations and Strengths

One limitation of this study is that it is cross-sectional, precluding anything but speculation about prediction of individual outcomes based on baseline findings. However, we hope that future longitudinal analyses of data from the POP project will allow us to investigate this. Another limitation is the amount of missing data, especially from the schizophrenia group. Naturally, more complete data would have been preferable, but is often difficult to achieve in this patient group for clinical and ethical reasons.

It is possible that some of the difference between the UHR and control group might be due to differences in education level. It is difficult to compare and control for this due to UHR subjects’ young mean age. However, school dropout before the end of mandatory schooling at the age of 16 is extremely rare in Norway. Matching as to years of education would thus not yield much additional information and might even be misleading when including both under 18s and adults in the control group. The young age of participants also precluded us from adequately measuring and comparing social cognition between groups, which might have yielded interesting results.

Owing to the criteria for prodromal syndrome including both brief psychosis-like experiences and loss of function over time, setting an accurate age of onset is a challenge for this group. This is especially the case for the genetic risk and deterioration syndrome group where it can be difficult to determine an exact starting point for this loss of function. It was decided in our research group that we were unable to create a reliable “DUP-like” variable for this group, although the inclusion of such a variable would have been of interest.

One of the main strengths of our study is the presence of a control group. Given that UHR patients are quite young, validated norms do not exist for all cognitive tests. Use of an age-matched control group allowed us to create coordinated norms to compare UHR youth to a schizophrenia group from a similar geographical area.

We have also attempted to overcome the challenge in UHR research that lack of access to health-care services for disadvantaged groups restricts recruitment, making the sample less representative. Our current project ran as part of Norway’s universal public health-care system, aiming to reach vulnerable youth across the catchment area. Despite the limitation on generalization caused by our relatively small sample size, we believe this strengthens our sample’s representativeness.

A further strength of our study is its placement within the Norwegian public health-care system, allowing us to recruit a wide range of UHR youth from a variety of socioeconomic backgrounds, without impacting on their opportunity to get treatment at the same cost without enrolling in our study. We believe that this makes our sample more representative of the general Norwegian UHR population.

## Conclusion

UHR subjects show impaired cognitive functioning in comparison with an age-matched healthy control group on speed of processing, working memory, and verbal learning, reasoning, and problem solving. Interestingly, spatial task performance appeared to be relatively unimpaired. UHR subjects performed better than a schizophrenia comparison group on speed of processing, but not in other measures. These findings highlight the importance of monitoring cognitive performance even in a prodromal phase of potential illness. Often less explored by clinicians than mood or attenuated psychotic symptoms, these symptoms may, in part, explain the high subjective distress reported by UHR groups, as cognitive impairment will impact both academic and social function.

## Data Availability

Data are from the Prevention of Psychosis project and the Bergen Psychosis Project 2 study, whose authors may be contacted *via* the corresponding author email (lissgoril@gmail.com). Owing to ethical restrictions in this project, data must be stored on dedicated secure hospital servers, even when anonymized, and may only be shared with research partners involved in the project. However, if anyone wishes to access the data, this may be granted after an application process.

## Ethics Statement

This study was carried out in accordance with the recommendations of Regional Committee for Medical and Health Research Ethics West and South East, with written informed consent from all subjects. All subjects gave written informed consent in accordance with the Declaration of Helsinki. The POP study protocol was approved by the Regional Committee for Medical and Health Research Ethics: South-East Committee [REK Sør-Øst C (ref. 2009/949)] while the BP 2 project protocol was approved by the Regional Committee for Medical and Health Research Ethics: West Committee [REK Vest (ref. 2010/3387)]. The combination of data from the two projects was separately approved by the West Committee (REK Vest 2018/342-3).

## Author Contributions

IJ, JJ, KB, RK, EJ, E-ML, MR, and LA contributed conception and design of the study as well as data collection. FF also contributed to the data collection. LA, E-ML and KB conducted the statistical analysis. LA wrote the first draft of the manuscript. All authors contributed to manuscript revision, read and approved the submitted version.

## Funding

The POP study has been financially supported by grant from the Norwegian Extra Foundation for Health and Rehabilitation (EXTRA funds no. 2011-1-27) and by grants from Health West, Norway (grant nos. 911508 and 911881). The BP2 study has been funded by the Norwegian research council (RCN) #213727 and the Western Norway Regional Health Authority #911820 and #911679.

## Conflict of Interest Statement

The authors declare that the research was conducted in the absence of any commercial or financial relationships that could be construed as a potential conflict of interest.
